# Self-reported physical fitness domains and perceptions of exercise and fatigue among childhood cancer survivors

**DOI:** 10.1016/j.jsampl.2026.100140

**Published:** 2026-05-19

**Authors:** Qamra Muaikel Alqahtani, David Mizrahi, Lauren Ha, Joanna E. Fardell, Claire E. Wakefield, David Simar, Richard J. Cohn, Paula Bray, Kimberley Docking, Elizabeth Dylke

**Affiliations:** a Rehabilitation Health Sciences Department, College of Applied Medical Sciences, King Saud University, Box 10219, 11433, Riyadh, Saudi Arabia; b Sydney School of Health Sciences, Faculty of Medicine and Health, University of Sydney, Sydney, New South Wales, 2006, Australia; c Saudi Commission for Health Specialties, Box 94656, 11614, Riyadh, Saudi Arabia; d The Daffodil Centre, University of Sydney, a Joint Venture with Cancer Council NSW, New South Wales, 1340, Australia; e Prince of Wales Clinical School, Faculty of Medicine and Health, UNSW Sydney, New South Wales, 2052, Australia; f Kids Cancer Centre, Sydney Children’s Hospital, Randwick, New South Wales, 2031, Australia; g School of Clinical Medicine, Discipline of Paediatrics, Faculty of Medicine and Health, UNSW Sydney, New South Wales, 2052, Australia; h Western Sydney Youth Cancer Service, Westmead Hospital, Sydney, New South Wales, 2145, Australia; i Sydney Medical School, Faculty of Medicine and Health, University of Sydney, Sydney, New South Wales, 2006, Australia; j Division of Quality of Life and Pediatric Palliative Care, Department of Pediatrics, Stanford University School of Medicine, Stanford, CA, 94305, USA; k School of Health Sciences, Faculty of Medicine and Health, UNSW Sydney, New South Wales, 2052, Australia; l Kids Research, The Sydney Children’s Hospitals Network, Sydney, New South Wales, 2145, Australia

**Keywords:** Paediatrics, Self-reported muscle strength, Self-reported running speed, Self-reported flexibility

## Abstract

**Background:**

Poor physical fitness and fatigue are prevalent long-term issues for childhood cancer survivors. This study aimed to determine associations between childhood cancer survivors’ self-reported physical fitness domains and their perceptions of links between exercise and fatigue.

**Methods:**

A cross-sectional survey assessed survivors' self-reported muscle strength, running speed and flexibility using The International Fitness Scale as well as survivors’ perceptions of exercise and fatigue. Non-parametric correlation tests were used.

**Results:**

One hundred and seven childhood cancer survivors aged 8–18 years participated at a median of six years post-completion of treatment. Poor muscle strength, running speed and flexibility were self-reported by 11%, 18% and 36% of survivors, respectively. Fatigue was perceived as a barrier to exercise by 50% of survivors, while 44% of survivors perceived that exercising reduced fatigue. Poor self-reported muscle strength was significantly associated with perceiving fatigue as a barrier to exercise (r_rb_ = −0.219) and with perceiving exercising to reduce fatigue (*T*_b_ = 0.192). Poor self-reported running speed was associated with perceiving fatigue as a barrier to exercise (r_rb_ = −0.199) and with perceiving exercising to reduce fatigue (*T*_b_ = 0.228).

**Conclusions:**

An assessment of self-reported physical fitness domains and perceptions of exercise and fatigue is needed among childhood cancer survivors to identify and support those who perceive poor physical fitness domains or believe that fatigue hinders their exercising.

**Implications for cancer survivors:**

Interventions that improve confidence in a childhood cancer survivor’s own ability to exercise should be investigated to improve re-engagement with exercise.

**What is known**

• Childhood cancer survivors benefit from regular participation in physical activity.
• However, poor physical fitness and high fatigue levels are common long-term issues for childhood cancer survivors, with fatigue being a commonly reported barrier to exercising.

**What is new**

• 46% and 41% childhood cancer survivors reported good muscle strength and running speed, respectively compared to their healthy peers.
• However, childhood cancer survivors who had poor self-reported muscle strength and running speed tended to perceive they could not exercise due to fatigue, while those with good self-reported physical fitness with their healthy peers believed that exercise made them less fatigued.

## 1 Introduction

Advances in cancer treatments have led to improvements in survival rates for children diagnosed with cancer [1]. However, many childhood cancer survivors are at risk of developing long-term adverse effects of cancer and its treatments, such as cardiovascular disease, in part due to decreased physical fitness, and ongoing issues with fatigue [[2], [3], [4], [5], [6]]. Physical fitness is defined as the characteristics related to performing physical activity, consisting of health- and skill-related components [7]. Health-related components of physical fitness include cardiorespiratory endurance, muscular endurance, muscle strength, body composition, and flexibility, while skill-related fitness includes speed, power, reaction time, agility, balance, and coordination [7]. Numerous studies have objectively measured different domains of physical fitness across varying age ranges and cancer diagnoses in childhood cancer survivors, showing low physical fitness [[8], [9], [10], [11], [12], [13], [14], [15], [16]]. However, objective assessment of physical fitness may not be feasible or practical, such as when the survivor cannot complete fitness testing due to maturity limitations, when qualified personnel or equipment are limited, or when there are insufficient resources available to assess a large sample size [8]. Several domains of physical fitness can be assessed using one brief self-reported tool, the International Fitness Scale (IFIS), which assesses five domains: overall fitness, cardiorespiratory fitness, muscle strength, running speed, and flexibility [17]. This tool, which has been shown to be valid in comparison to objective measurement of physical fitness in healthy children [17,18], has found that considerable proportions of children treated for cancer reported poor physical fitness across all domains during the first weeks following a cancer diagnosis [19] and after completion of cancer treatment [20,21]. However, little is known about whether children surviving cancer perceive each domain of their physical fitness, such as muscle strength, running speed, or flexibility, similarly. As perceived fitness may impact on an individual’s willingness to engage with things like physical activity programs, it is worthwhile investigating if there is a relationship between self-belief in fitness domains and factors such as fatigue.

Better physical fitness and regular participation in physical activity are associated with several health benefits for childhood cancer survivors, including improved quality of life, reduced risk of cardiovascular disease, and decreased fatigue [22]. However, fatigue is a prevalent distressing problem among this population [23,24], and survivors of childhood cancer also reported fatigue as the most frequent barrier to physical activity [[25], [26], [27]]. Cancer-related fatigue is a subjective feeling of physical, cognitive, and/or emotional tiredness or exhaustion related to cancer or treatments of cancer, which is bothersome, ongoing, non-proportional to recent activity and hinders normal functioning [28]. Exercise and physical activity interventions are safe and effective in reducing fatigue in childhood cancer patients and survivors [[29], [30], [31]], and evidence-based physical activity is being more widely promoted to be included as a component of standard care for this population [32,33]. However, in a study of 102 childhood cancer survivors, fatigue is the highest reported barrier to exercising (52%), as well as the least commonly reported facilitator to engage in exercise (44%) [34]. It is currently unknown whether an association exists between a childhood cancer survivor’s assessment of their physical fitness levels and their perceptions of the role of exercise with fatigue.

Understanding childhood cancer survivors perceived physical fitness domains as well as their perceptions of exercise and fatigue would contribute evidence required to promote physical activity after completing cancer treatment. The aims of this study were therefore to:

1 Examine survivors' self-reported physical fitness domain levels and determine the association between three self-reported physical fitness domains, i.e., muscle strength, running speed and flexibility,
2 Investigate survivors' perceptions of exercise and fatigue and define the association between those perceptions, and
3 Determine the associations between survivors' self-reported physical fitness domain levels and their perceptions of exercise and fatigue.

## 2 Methods

The current study is a secondary analysis of cross-sectional data collected during two previous studies [20,34]. These primary studies aimed to investigate physical activity levels, facilitators and barriers of physical activity, and aerobic fitness of childhood cancer survivors [34] and examine their perceptions of physical activity and their cardiovascular fitness levels [20]. In the primary studies, self-reported data were collected using a child baseline questionnaire about exercise reported in the appendix of [35]. The child baseline questionnaire involved a modified version of the International Fitness Scale (IFIS) [17], with data related to the domain of cardiovascular fitness having already been reported in Ha et al. (2021) [20]. The child baseline questionnaire also contained two questions about survivors’ perceptions of several problems (barriers) and reasons (facilitators) to engage in exercise, including fatigue, which has seen previously published in Mizrahi et al. (2020) [34]. The child baseline questionnaire was pilot tested with 10 healthy children for readability and modified based on their feedback [35].

The current study used responses to three questions of the International Fitness Scale (IFIS) about survivors' self-reported physical fitness domains, which have not been published, in addition to responses of survivors’ perception of exercise and fatigue, which were reported in one of the primary studies [34]. This study followed the Strengthening the Reporting of Observational Studies in Epidemiology (STROBE) guidelines for cross-sectional studies [36].

### 2.1 Participants and procedures

Participants were recruited from the Kids Cancer Centre at Sydney Children’s Hospital, Australia, between September 2017 and March 2020. Eligible participants for the purpose of the primary studies were children aged 8–18 years at the time of the study, had completed cancer treatment at least one year earlier, and could communicate in English. Any potential participant who had a medical contraindication to cardiopulmonary exercise testing (e.g., uncontrolled cardiac disorder, uncontrolled asthma, cognitive impairment, severe peripheral neuropathy, paralysis, amputation, or prosthesis) was excluded after confirmation by their treating consultant. Potential participants were identified by a researcher from the primary studies and nursing staff through clinic lists, and then the eligibility of the potential participants was confirmed by their treating consultant. Parents of eligible survivors were contacted by e-mail or phone, and informed consent was obtained from the parents of survivors. Survivors and their parents were asked to complete their self-reported questionnaire online or using a printed copy with a postage-paid envelope sent by mail. For younger children, i.e., 8–10 years old, assistance was permitted when required, limited to practical support such as reading questionnaire items aloud or clarifying instructions. Parents or research staff did not interpret items, suggest responses, or answer questions on behalf of the child. Older children and adolescents completed questionnaires independently. Ethical approvals for the primary studies were received from The Sydney Children’s Hospitals Network Human Research Ethics Committee (LNR/16/SCHN/403 and HREC/18/SCHN/471). The research was conducted in accordance with the National Health and Medical Research Council (Australia) standards.

### 2.2 Measures

#### 2.2.1 Demographics and clinical history

The child’s age, gender, and cancer-related clinical data, including initial cancer diagnosis, date of cancer diagnosis, treatment received (e.g., chemotherapy received: yes/no/unsure), and time since completion of cancer treatment, were obtained from their parents via a questionnaire. A digital stadiometer was utilised to measure the child’s height, and survivors were weighed to determine their Body Mass Index (BMI). Height and weight were taken routinely by a clinical nurse for routine follow up assessment. The study survey assessment was completed on the same day as the follow up appointment.

#### 2.2.2 Self-reported physical fitness domain levels

Three questions from the International Fitness Scale (IFIS) related to muscle strength, running speed and flexibility [17] were used in this study. The IFIS measures the children’s self-reported physical fitness by asking them to rate their physical fitness in comparison with their friends on a 5-point Likert scale (1 = very poor to 5 = very good) [17]. The IFIS has shown good test-retest reliability in healthy children aged 9.0–17.5 years [17,37]. Minor modifications to the wording of the IFIS were carried out to make the questions more accessible for children based on participants' feedback. For example, altering “Your muscular strength is” with “My muscle strength is (e.g., how strong you are)” and replacing “Your speed/agility is” with “My running speed is (e.g., how fast you can run),” was done to clarify the questions for children [35]. The five responses for each domain of the IFIS were trichotomised to “poor/very poor”, “average”, and “good/very good” for this analysis.

#### 2.2.3 Perceptions of exercise and fatigue

A survivor was considered to believe that fatigue was a barrier to exercise if they selected “Too tired” as a response option for the question: “Do you ever have problems exercising because of the following:” For the purpose of this study, this will be referred to as “perceiving fatigue as a barrier to exercise.”

Survivors were also asked to answer the question “How do you feel about the following reasons to do exercise?” using a 5-point Likert scale, i.e., strongly agree, agree, neither agree/disagree, disagree, and strongly disagree. Survivors were considered to believe that doing exercise made them less fatigued if they rated “It makes me feel less tired” as “strongly agree” or “agree” options. This will be referred to as “perceiving exercising to reduce fatigue” in the current study. The five responses for perceiving exercising to reduce fatigue were trichotomised to “agree/strongly agree”, “neutral”, and “disagree/strongly disagree”.

### 2.3 Data analyses

#### 2.3.1 Computing and classifying Body Mass Index (BMI)

BMI of survivors was calculated by dividing their weight in kilograms by their height in metres squared. Classification of survivors' BMI was then computed using paediatric-specific cut-off points for grouping BMI scores by sex for exact age in years for Australian children [38]. The classification of survivors’ BMI was according to the International Obesity Task Force for childhood BMI cut-offs for underweight, overweight, and obesity [39,40]. BMI scores were grouped into four categories: underweight, normal weight, overweight, and obese.

#### 2.3.2 Statistical analyses

Demographic and clinical characteristics of participants were analysed with descriptive statistics, including medians (ranges and interquartile ranges) and frequencies. Frequencies were used to summarise the study variables. Non-parametric statistical tests were used because the study’s variables were categorical data. The associations between ordinal variables, i.e., self-reported muscle strength, self-reported running speed, self-reported flexibility, and perceiving exercising to reduce fatigue, were examined with Kendall’s coefficient of rank correlation tau-sub-b, with an estimation of Confidence Interval (CI) based on Fisher’s r-to-z transformation. A rank–biserial correlation coefficient was used for exploring the associations between a binominal variable, i.e., perceiving fatigue as a barrier to exercise, and ordinal variables, with the estimation of the CI based on Fisher’s r-to-z transformation and the standard error based on the method proposed by Fieller, Hartley and Pearson [41]. All statistical analyses were conducted using IBM SPSS Statistics for Windows, version 28.0 (IBM Corp., Armonk, NY, USA). All tests were two-sided, and statistical significance was set at *P* < 0.05.

## 3 Results

### 3.1 Participants’ characteristics

Primary data included 117 survivors, with a response rate of 86.8% [20]. However, data from 10 survivors were removed from the analysis due to missing data for survivors' variables (*N* = 8) or having non-cancer diagnoses, e.g., Fanconi anaemia (*N* = 2). This study, therefore, involved 107 survivors. The majority of survivors were males (59.8%), with a median age of 14 years old (Table 1). Twenty-two percent and 13.1% of survivors were overweight and obese, respectively. The most frequent cancer diagnoses were acute lymphoblastic leukaemia (47.7%), followed by Hodgkin’s lymphoma (12.1%). Most survivors received more than one type of cancer treatment, with majority of survivors receiving chemotherapy (96.3%) followed by surgery (68.2%). The time since completion of cancer treatment ranged from 1.1 to 14.4 years, with a median of 5.5 years.

**Table 1:** Characteristics of participants.

Survivor characteristic	*N* = 107
Gender (males: Females): N (%)	64 (59.8%): 43 (40.2%)
Age at study (years): Median (IQR)	14 (11–16)
Age at diagnosis^a^ (years): Median (IQR)	4 (2.0–7.3)
BMI (kg/m^2^)^b^: Median (IQR)	19.9 (17.4–23.1)
*BMI classification*^b^	N (%)
Underweight	16 (15.0%)
Normal weight	51 (47.7%)
Overweight	24 (22.4%)
Obese	14 (13.1%)
*Cancer diagnosis*	N (%)
Acute lymphoblastic leukaemia	51 (47.7%)
Hodgkin’s lymphoma	13 (12.1%)
Wilms' tumour	9 (8.4%)
Neuroblastoma	6 (5.6%)
Acute myeloid leukaemia	5 (4.7%)
Brain tumour	3 (2.8%)
Hepatoblastoma	3 (2.8%)
Non- Hodgkin’s lymphoma	3 (2.8%)
Rhabdomyosarcoma	3 (2.8%)
Acute promyelocytic leukaemia	2 (1.9%)
Ovarian cancer	2 (1.9%)
Other cancer diagnosis^c^	7 (6.5%)
*Treatment received*^b^^,^^d^	N (%)
Chemotherapy	103 (96.3%)
Surgery	73 (68.2%)
Radiotherapy	28 (26.2%)
Bone marrow transplant	24 (22.4%)
Years since treatment completed^e^: Median (IQR)	5.5 (2.1–9.5)
1-<5 years: N (%)	47 (43.9%)
5–10 years: N (%)	32 (29.9%)
>10 years: N (%)	21 (19.6%)

Abbreviations: N: Number, %: Percentage, IQR: Interquartile Range (25%–75%), BMI: Body Mass Index.

a *Missing data: N* = 5 (4.7%).

b *Missing data: N* = 2 (1.9%).

c Other cancer diagnoses (*N* = 1 each, 0.9%): Bone sarcoma, chronic myeloid leukaemia, clear cell sarcoma of the kidney, germ cell testicular tumour, inflammatory myofibroblastic tumour, primitive neuroectodermal tumour, soft tissue sarcoma.

d Most survivors received more than one type of cancer treatment.

e *Missing data: N* = 7 (6.5%).

### 3.2 Self-reported physical fitness domain levels

**Fig. 1:**
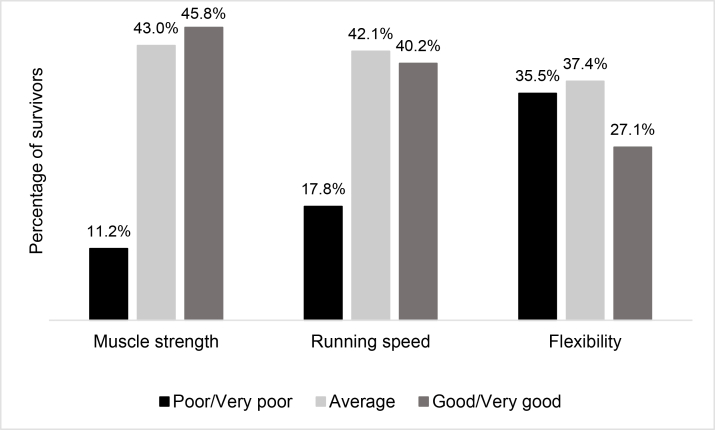
Survivors' self-reported physical fitness domain levels (*N* = 107).

Approximately half (45.8%) of the survivors in the current study perceived their muscle strength was “good” or “very good” relative to their peers (Fig. 1). Running speed and flexibility were most commonly perceived as ‘average’ by 42.1% and 37.4% of survivors, respectively. However, 35.5%, 17.8% and 11.2% of survivors perceived their flexibility, running speed, and muscle strength, respectively, to be “poor” or “very poor” (Fig. 1).

Survivors' self-reported muscle strength had a moderate positive association with their self-reported running speed (*T*_b_ = 0.305, 95% CI: 0.185–0.416, *P* < 0.001) and a weak positive association with their self-reported flexibility (*T*_b_ = 0.193, 95% CI: 0.067–0.312, *P* = 0.029). However, survivors’ self-reported running speed was not associated with their self-reported flexibility (*T*_b_ = 0.143, 95% CI: 0.016–0.265, *P* = 0.102).

### 3.3 Perceptions of exercise and fatigue

Nearly half of survivors perceived fatigue as a barrier to exercise (*N* = 53/107; 49.5%). Regardless of whether they perceived fatigue as a barrier to exercise, 44% (*N* = 46/105) believed that exercise reduced fatigue, whereas 34% (*N* = 36/105) did not believe that it did, with the rest of the participants neutral on this idea (*N* = 23/105; 22%). There was no association between survivors’ perception of fatigue as a barrier to exercise and their perception of exercising to reduce fatigue (r_rb_ = −0.105, 95% CI: −0.296 – 0.094, *P* = 0.288, *N* = 105).

### 3.4 Associations between self-reported physical fitness domain levels and perceptions of exercise and fatigue

Survivors' perception of fatigue as a barrier to exercise was negatively weakly associated with their self-reported muscle strength and with their self-reported running speed (Table 2). Survivors' perception of exercising to reduce fatigue was positively weakly associated with their self-reported running speed and with their self-reported muscle strength (Table 2). However, survivors’ self-reported flexibility was not associated with their perception of fatigue as a barrier to exercise or with their perception of exercising to reduce fatigue (Table 2).

**Table 2:** Associations between physical fitness domains and perceptions of exercise and fatigue.

Survivors' self-reported physical fitness domains	Perceptions of exercise and fatigue	
	*Fatigue as a barrier to exercise* (*N* = 107)	*Exercising to reduce fatigue* (*N* = 105^a^)
*Muscle strength* (*N* = 107)	r_rb_ = **-0.219** 95% CI: −0.397 to −0.025 *P* = 0.023	*T*_b_ = **0.192** 95% CI: 0.065–0.312 *P* = 0.032
*Running speed* (*N* = 107)	r_rb_ = **-0.199** 95% CI: −0.380 to −0.004 *P* = 0.040	*T*_b_ = **0.228** 95% CI: 0.103–0.346 *P* = 0.010
*Flexibility* (*N* = 107)	r_rb_ = 0.029 95% CI: −0.167 – 0.223 *P* = 0.767	*T*_b_ = 0.163 95% CI: 0.035–0.285 *P* = 0.064

Abbreviations: N: Number, CI: Confidence Interval: lower–upper.

a Missing data from two participants.

## 4 Discussion

The current study showed that some (27–36%) of childhood cancer survivors felt their muscle strength, running speed, and flexibility were good or very good compared to their peers despite the significant treatments they had completed. However, many (11–36%) survivors perceived that they were weaker, slower, and less flexible than their peers, even at a median of 5.5 years since completion of cancer treatment. Furthermore, many also still perceived fatigue as a barrier to exercise and were more likely to do so if they had poor self-reported muscle strength and running speed. In contrast, those who reported good muscle strength and running speed reported that exercise made them less fatigued. This study raises important information about the links between self-belief in physical fitness, fatigue and physical activity participation.

The findings from this study on how children perceive their muscle strength, running speed, and flexibility after completion of cancer treatment are supported by a larger study, which also found that childhood cancer survivors have mixed beliefs about their own physical fitness [21]. Benzing et al. (2021) [42] also found that childhood cancer survivors reported comparable strength and lower flexibility than controls, which aligns with survivors' perception of their muscle strength and flexibility in the current study. However, some research has not found any association between self-reported strength and flexibility [42] or between hand grip strength and running time measured by shuttle run test among childhood cancer survivors [43]. The current study revealed that survivors who perceived their strength was good were more likely to perceive that they had good running speed and flexibility. Running is a fundamental movement skill for children that is positively associated with their participation in physical activity [44] and health-related quality of life [21]. However, children may consider their running ability and physical fitness in general in terms of their ability to play and keep up with their peers’ recreational activities rather than through engaging in structured physical activity. As less than 28% of children treated for cancer have been reported to achieve the recommended 60 minutes of moderate to vigorous activity per day [20,45], the running ability of childhood cancer survivors, as well as their ability to play in an unstructured environment, is an important survivorship consideration that may require further investigation.

In line with the current literature, this study showed that half of the childhood cancer survivors perceived fatigue as a barrier to exercise [[25], [26], [27],^46^,^47^]. While participants in this study frequently believed that exercising made them less fatigued, there was no association between perceptions of exercise and fatigue. It is possible that survivors who did not believe fatigue was a barrier to exercise or did not perceive doing exercise to reduce fatigue, may not have experienced fatigue themselves. It is also possible that this group of survivors may lack knowledge of the benefits of exercise for fatigue. Despite the known benefits of exercise to reduce fatigue for childhood cancer patients and survivors [[29], [30], [31]], research indicates that some patients reported lack of education received about these benefits from medical professionals [48], with some oncologists perceived lack of knowledge/resources as a barrier to counselling survivors to be physically active [49]. Fundamentally, cancer-related fatigue is a complex and multifaceted phenomenon [50], with many contributing factors beyond physical activity or self-perception of fitness. For example, a recent meta-analysis has shown that objectively measured muscle strength was inversely associated with fatigue in adults diagnosed with cancer [51], demonstrating the complexity of associations between physical fitness and fatigue. The current study also found that survivors' self-reported muscle strength was associated with their perceptions of exercise and fatigue. Muscle strength, therefore, might play a mediating role in the relationship between exercise and fatigue. Having good muscular strength might promote the ability to engage in exercise and correspondingly feel less fatigued. This requires more investigation in childhood cancer survivors’ populations.

While this study raises some useful findings, it should be acknowledged that the aim of the primary studies [20,34] was not to specifically explore the perceptions of exercise and fatigue. Furthermore, participants were recruited from one setting and had to be cleared to undertake physical activity, both of which could impact the generalisability of this study’s findings to a larger population. Finally, although fatigue is often a multi-faceted experience, for simplicity a single measure of fatigue was used in this study, which doesn’t capture all aspects of fatigue, such as severity. However, the current study is the first to determine the association between self-reported physical fitness domains and perceptions of exercise and fatigue among childhood cancer survivors, which requires further investigation as those survivors who perceive they have poor muscle strength and running speed may be more likely to consider fatigue a barrier to exercise. In a busy clinical environment, it can be difficult from a time and resources perspective to routinely assess physical fitness in children with cancer. This study utilised a simple brief tool to understand children’s perspective of their physical fitness domains compared with their peers, which could be utilised in a busy clinical environment. Further prospective mixed methods research might help understand the link between exercise and fatigue from childhood cancer survivors' perspectives.

## 5 Conclusion

Children who have been treated for cancer have mixed beliefs about their own physical fitness, which appears to correlate with their beliefs around physical activity and fatigue. While interventions to encourage physical activity often focus on the programs themselves, these findings suggest that assessing and encouraging the children’s self-belief in their fitness may be a useful adjunct to programs in the future. This may be particularly important for encouraging long-term engagement in physical activity programs, which will benefit children as they age and recover from the treatment they undertook for cancer.

## Consent to participate

Written informed consent was obtained from the parents of participants prior to study enrolment.

## Author contributions

David Mizrahi, David Simar, Joanna E. Fardell and Claire E. Wakefield contributed to the original research conceptualization. Material preparation and data collection were performed by David Mizrahi and Lauren Ha. Data analysis was conducted by Qamra Muaikel Alqahtani and Elizabeth Dylke. The first draft of the manuscript was written by Qamra Muaikel Alqahtani and edited by Elizabeth Dylke. All authors reviewed and edited the manuscript, and all authors approved the final manuscript.

## Ethics approval

The study was approved by The Sydney Children’s Hospitals Network Human Research Ethics Committee (LNR/16/SCHN/403 and HREC/18/SCHN/471).

## Data availability

The datasets generated during and/or analysed during the current study are available from the corresponding author on reasonable request.

## Consent for publication

Written informed consent for publication of de-identified data was obtained from the parents of participants.

## Funding

The Behavioural Sciences Unit at Sydney Children’s Hospital is supported by the Kids with Cancer Foundation. Author D.M. was supported by an Australian Government Research Training Program Scholarship. Authors L.H. and J.E.F. were supported by the Kids' Cancer Project. Author C.E.W. was supported by the NHMRC of Australia (APP2008300). Author Q.M.A. had a PhD scholarship from the Saudi Arabian Cultural Mission to study in Australia.

## Declaration of competing interest

The authors declare that they have no known competing financial interests or personal relationships that could have appeared to influence the work reported in this paper.
